# Importance of Interaction between Integrin and Actin Cytoskeleton in Suspension Adaptation of CHO cells

**DOI:** 10.1007/s12010-015-1945-z

**Published:** 2015-12-17

**Authors:** Christa G. Walther, Robert Whitfield, David C. James

**Affiliations:** Department of Molecular Biology and Biotechnology, University of Sheffield, Firth Court, Western Bank, Sheffield, S10 2TN UK; Department of Applied Sciences and Health, Coventry University, James Starley Building, Coventry, CV1 5FB UK; Chemical and Biological Engineering, University of Sheffield, Sir Robert Hadfield Building, Mappin Street, Sheffield, S1 3JD UK

**Keywords:** Chinese hamster ovary cells, Suspension adaptation, Cell surface proteins, Integrin signalling, Cell-to-extracellular matrix interaction, Actin cytoskeleton

## Abstract

The biopharmaceutical production process relies upon mammalian cell technology where single cells proliferate in suspension in a chemically defined synthetic environment. This environment lacks exogenous growth factors, usually contributing to proliferation of fibroblastic cell types such as Chinese hamster ovary (CHO) cells. Use of CHO cells for production hence requires a lengthy ‘adaptation’ process to select clones capable of proliferation as single cells in suspension. The underlying molecular changes permitting proliferation in suspension are not known. Comparison of the non-suspension-adapted clone CHO-AD and a suspension-adapted propriety cell line CHO-SA by flow cytometric analysis revealed a highly variable bi-modal expression pattern for cell-to-cell contact proteins in contrast to the expression pattern seen for integrins. Those have a uni-modal expression on suspension and adherent cells. Integrins showed a conformation distinguished by regularly distributed clusters forming a sphere on the cell membrane of suspension-adapted cells. Actin cytoskeleton analysis revealed reorganisation from the typical fibrillar morphology found in adherent cells to an enforced spherical subcortical actin sheath in suspension cells. The uni-modal expression and specific clustering of integrins could be confirmed for CHO-S, another suspension cell line. Cytochalasin D treatment resulted in breakdown of the actin sheath and the sphere-like integrin conformation demonstrating the link between integrins and actin in suspension-adapted CHO cells. The data demonstrates the importance of signalling changes, leading to an integrin rearrangement on the cell surface, and the necessity of the reinforcement of the actin cytoskeleton for proliferation in suspension conditions.

## Introduction

Chinese Hamster ovary cells (CHO) are one of the earliest established mammalian cell lines [1]. In standard cell culture conditions, which include foetal calf serum [2], this adherent epithelial cell line shows fibroblast growth patterns [3]. CHO cells have been used since 1986 as production vehicle [4, 5] in large-scale biopharmaceutical production due to their capability to produce high protein concentrations, a good consistency in product characteristics [5, 6] in combination with their ability to produce highly N-glycosylated proteins [7, 8].

Therefore, CHO cells are the workhorse in many biopharmaceutical processes [4, 9] and used to produce e.g. monoclonal antibodies and anti-inflammatory cytokines. The market for biopharmaceutical products grows consistently [10, 11], and the growing demand for those therapeutic products will only be met by optimisation of productivity and increasing achievable viable cell densities of the cell lines used [12]. Optimisation of a single clone used in production is hence a key factor for a production of a stable production process which delivers appropriate amounts of product at an acceptable production cost [6].

Animal component-free media are preferred as those culture conditions reduce the risk of virus introduction or of other transmissible agents. In addition, upstream processing of secreted product is simplified when no serum is supplemented. Hence, biopharmaceutical production processes usually use a single-cell suspension reactor together with chemically defined, animal component-free media [13]. As wild-type mammalian cell strains such as CHO cells usually grow adherently and require serum supplementation, establishing a cell line capable of proliferation in a suspension culture without serum supplementation is one of the major steps in the development of a biopharmaceutical production process [14]. Resulting suspension-adapted cells are usually able to grow without serum to high densities yielding production titres of above 5 g/ml [6].

The adaptation process itself is a long multistep process [14] where cells are first adapted to suspension growth and serum is subsequently reduced. The success rate of this process is below 100 %; sometimes, additional growth factors are included to achieve suspension adaptation [3, 15]. So far, little is known about the changes in the cells itself that allow for successful adaptation to a new environment. It is known that interruption of cell surface protein signalling, such as the loss of integrin signalling due to disruption of extracellular matrix-cell contact as introduced by serum withdrawal, leads to a specific form of apoptosis called anoikis [16, 17]. Hence, changes in membrane protein expression and cell metabolism need to occur during the adaption process to allow for successful proliferation in the new environment [18].

A deeper understanding of the changes that cells undergo during suspension adaptation will enhance cell engineering possibilities [19] and also allows faster selection methods for the best suspension cell lines and hence allows to speed up cell line development.

In this study, cell surface protein expression of adherently growing CHO cells (CHO-AD) and suspension-adapted CHO cells (proprietary cell line CHO-SA) has been investigated using antibody staining in combination with flow cytometry and confocal microscopy to reveal differences in expression and distribution of cell surface proteins. As integrins, a subgroup of membrane proteins, showed a conserved expression and a specific distribution in suspension-adapted CHO cells, the underlying actin cytoskeleton as a major intracellular interaction partner of integrins was studied using the same methods. The conserved expression and specific distribution of integrins were confirmed for another suspension cell line, CHO-S (FreeStyle^TM^ from Invitrogen).

## Material and Methods

### Cell Lines and Cell Culture

Three cell lines were used: adherent CHO clone CHO-AD/CCL-61; CHO-SA, a proprietary suspension cell line from Pfizer (Andover, USA) and the suspension cell line FreeStyle^TM^ CHO-S from Invitrogen. Cell culture conditions were 5 % CO_2_, 37 °C and for suspension culture 140 rpm in vented 25-ml Erlenmeyer shake flasks (Corning, Surrey, UK). For CHO-SA, proprietary media from Pfizer (Andover, USA) containing 10 mg/ml insulin was used. CHO-S were cultured in CD-CHO medium (Invitrogen, Paisley, UK) supplemented with 8 mM glutamine (Sigma-Aldrich, St. Louis, USA). Adherent cell cultures were split using 0.05 % trypsin/EDTA (Gibco, Paisley, UK). CHO-AD were cultured in Ham’s modified F-12K medium (LGC, Teddlington, UK) supplemented with 10 % foetal bovine serum (FBS) (Biosera, Ringmer, UK). The same conditions were used for all adherent growth experiments. Measurements of cell density and cell viability were performed using the Vi-Cell XR Cell Viability Analyser (Beckmann Coulter, High Wycombe, UK).

### Cell Fixation for Staining

Adherent cells were harvested using phosphate-buffered saline (PBS) with 2 mM EDTA (Fisher Scientific, Lougborough, UK). For microscopy, adherent cells were grown on cover slips which had been prepared by incubation with 1 ml of FBS overnight. Cover slips with cells were washed twice and fixed with 1 % paraformaldehyde (PFA; 95 %, Sigma-Aldrich, Gillingham, UK) for 20 min at 21 °C. Non-adherent cells were spun down and washed before fixation with 1 % PFA (1 ml/5 × 10^6^ cells), incubating at 4 °C for 20 min.

## Flow Cytometry

### Actin Staining

Actin was stained using Alexa 546 phalloidin (Molecular Probes, Paisley, UK), prepared according to datasheet. For permeabilisation, 2 × 10^6^ fixed cells were washed twice with PBS and 50 μl 0.1 % Triton X-100 (Sigma-Aldrich, Gillingham, UK) were added, followed by incubation at 21 °C for 3 min. Cells were washed twice with PBS (200 g, 5 min) before adding 200 μl 1 % bovine serum albumin (BSA; microbiological grade, Fisher Scientific, Loughborough, UK) in PBS with 5 μl of the prepared phalloidin stock solution. The cells were incubated for 20 min at 21 °C and washed once with PBS. Cells were stored for a maximum of 24 h in 1 % BSA/PBS buffer.

### Antibody Staining

Antibodies used: integrin beta 1 clone 9EG7, integrin alpha 1 clone Ha31/8, anti-rat IgG2a PE clone RG7/1.30, Streptavidin-FITC, all from BD Biosciences (Oxford, UK) and integrin alpha 4 clone R1-2, CD56 clone AF12-7H3, CD44 clone DB105, CD324 clone 67A4, all Miltenyi Biotec (Bergisch Gladbach, Germany).

Integrin alpha 1 antibody biotinylation: 1 mg Sulfo-NHS-S-S-Biotin was dissolved in 200 μl PBS/EDTA. Integrin alpha 4 antibody was diluted 1 to 5. To 20 μl of antibody dilution, 2 μl of the prepared biotin solution was added and incubated over night at 21 °C while mixing.

Cell staining: 2 to 2.5 × 10^6^ cells in 50 μl PBS/0.2 mM EDTA/0.5 % BSA buffer. For integrin beta 1, antibody titre was 1 to 21; for all other clones including biotinylated integrin alpha 1, antibody titre was 1 to 6; incubation time was 45 min (30 min for integrin alpha 1) at 4 °C. Before secondary staining, two washes were performed. As secondary antibody, for integrin beta 1, mouse anti-rat IgG2a PE (1 to 100) and for integrin alpha 4, Streptavidin-FITC [1–25] were used, incubation time was 15 min at 4 °C. All other clones were direct conjugates to either PE or APC. Stained cells were stored for a maximum of 24 h in 1 ml of PBS/0.2 mM EDTA/0.5 % BSA buffer.

Flow cytometric analysis was performed using a FacsCalibur (BD Biosciences, Oxford, UK). Flow cytometry data was analysed using FlowJo 7.6.5 (Freestar Inc., USA).

## Confocal Microscopy

### Actin Staining

Staining conditions were the same as for flow cytometry. For suspension cells, cell pellets were dissolved in two drops of PBS and mounted on a glass slide; all cells were mounted using approximately 50 μl ProLong Gold antifade (Invitrogen, Paisley, UK).

### Antibody Staining

Additional antibodies used: Alexa Fluor 488 goat anti-mouse IgG2b (Molecular Probes, Paisley, UK), anti-Armenian and Syrian Hamster IgG FITC (BD Biosciences, Oxford, UK), integrin alpha 4 biotin (Miltenyi Biotec, Bergisch Gladbach, Germany). Cell staining: Either a cover slip with adherent cells or 2.5 × 10^6^ suspension cells were washed twice with PBS, and 50 μl 1 % BSA in PBS was added. Either integrin beta 1 antibody (titre 1 to 21), integrin alpha 1 antibody [1–6] or integrin alpha 4 biotin [1–6] was added; incubation was for 45 min at 4 °C followed by two washes. For secondary antibody staining, 50 μl 1 % BSA buffer with either Alexa Fluor 488 goat anti-mouse IgG2b or anti-Armenian and Syrian Hamster IgG FITC (1 to 100) or streptavidin-FITC [1–25] were added and incubated for 15 min at 4 °C. Cells were washed once; for suspension cells, pellets were dissolved in two drops of PBS and mounting was done on glass slides using approximately 50 μl ProLong Gold antifade (Invitrogen, Paisely, UK).

For confocal microscopy analysis, an inverted Zeiss (Oberkochen, Germany) LSM510 Meta Confocal Microscope was used. Mounted cell samples were analysed using a plan apochromat 63×/1.4 oil DIC objective with excitation provided by an argon laser. FITC/Alexa 488 excitation used 488 nm and Alexa 546 excitation 514 nm. Emission was collected for FITC/Alex 488 using a band pass 500-550 IR filter and for Alexa 546 using a long pass 560 filter. All images were analysed using the Zeiss LSM Image Browser Version 4,2.0,121 and Image J.

### Treatment with Cytochalasin D

Cells were grown in adherent conditions for 4 days. After harvest using trypsin/EDTA and transfer into adherent growth media with FBS, cells were seeded in propriety suspension medium with of 0.4 × 10^6^ cells/ml ensuring that no FBS was transferred. Cells were treated with 5 μM cytochalasin D in EtOH; control samples were treated with the equivalent amount of EtOH; incubation with cytochalasin D was for 8 h before a set of samples was analysed for actin and integrin beta 1. Another subset of samples was spun down to remove cytochalasin D and resuspended in propriety suspension medium, readjusting the seeding density to 0.4 × 10^6^/ml. Cells were cultured in suspension mode for 18 h and analysed again with regard to integrin beta 1 and actin conformation. In addition, cell densities and viability were analysed.

### Western Blotting

Twenty-five micrograms of protein from cells lysed with RIPA buffer containing HALT Protease and Phosphatase Inhibitor Cocktail (Thermo Scientific) was used for western blotting. Samples were run on NuPAGE 10 % Bis-Tris gels (Invitrogen) using MOPS buffer (Invitrogen) under reduced conditions. Running condition were 175–200 V for approximately 90 min. Gels were then electro-transferred to a PVDF membrane using an iBlot dry blotting system (Invitrogen). After washing with TBS for 5 min, blocking was performed for 1 h at 21 °C followed by further three washes at 5 min. Incubation with the primary antibody phospho-focal adhesion kinase (FAK; Tyr397) (clone D20B1) from Cell Signaling Technology was performed in 10 ml of primary antibody dilution buffer with gentle agitation overnight at 4 °C. After three washes, the membrane was incubated with the species-appropriate horseradish peroxidise (HRP)-conjugated secondary antibody (1:2000 dilution) and HRP-conjugated anti-biotin antibody (1:1000) to detect biotinylated protein markers in 10 ml of blocking buffer for 1 h at room temperature, followed by three 5-min washes. For detection, the membrane was incubated with ImmobilonTM Western Chemiluminescent HRP substrate (Millipore) prepared as per manufacturer’s instructions for 5 min. Analysis via imaging and densitometry was done using an ImageQuant^TM^ RT ECLTM system (GE Healthcare) together with the ImageQuant TL 1D gel analysis software (GE Healthcare).

## Results and Discussion

### Different Adapted CHO Cell Lines—Variation in Cell Growth

**Fig. 1:**
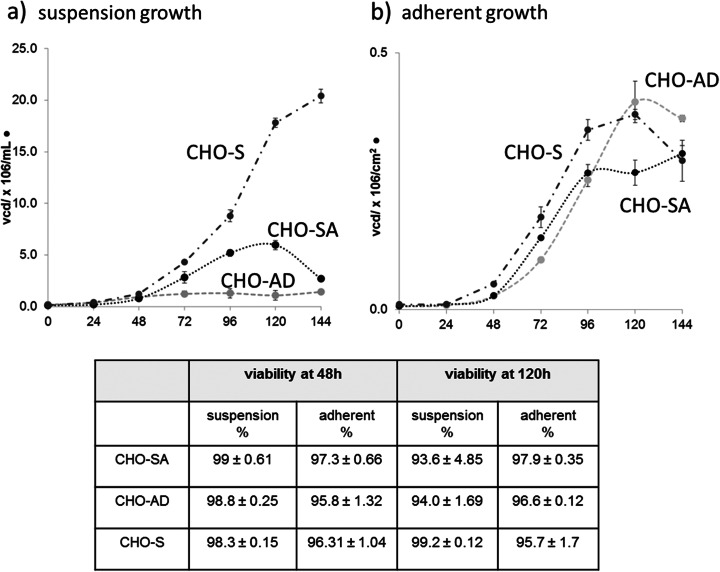
*Environmental dependence of growth behaviour for CHO-SA, CHO-AD and CHO-S.* Viable cell densities were determined for all three cell lines in **a** suspension and **b** adherent growth conditions. *Black dotted lines* are for CHO-SA, *dark grey dashed lines* for CHO-S and *mid grey dashed* for CHO-AD. Viable cell densities (vcd) and viability were measured over a period of 6 days (*n* = 3). Diameters of harvested cells were determined separately, yielding 14.47 ± 0.31 μm for CHO-AD, 14.39 ± 0.72 μm for CHO-SA and 16.05 ± 1.12 for CHO-S

To analyse the proposed difference in the cell surface, protein expression-related CHO cell lines were chosen with CHO-AD being the adherent parental clone to the suspension cell line CHO-SA and the CHO-S cell line. CHO-SA has been generated in the usual way of using stepwise adaptation to suspension growth, finally switching to chemically defined serum-free media. The commercially available cell line CHO-S [20] has undergone additional selection to provide a high viable cell density in suspension. Figure 1 shows that CHO-SA and CHO-S proliferate in suspension and adherent growth conditions whereas the parental clone shows very low proliferation in suspension conditions with the growth rate reduced by 40 % and maximum viable cell density reduced by nearly 80 % in comparison with the CHO-SA. CHO-S achieves a three times higher viable cell density in suspension compared to CHO-SA. All cell lines show similar growth characteristics in adherent growth conditions.

### Expression of Specific Cell Surface Proteins

To analyse the proposed difference in the cell surface proteome, antibodies binding to surface proteins involved in cell-to-cell contact and extracellular matrix interaction were tested on CHO cells. This generated a set of cross-reactive antibodies allowing both subgroups of surface proteins to be tested. The first group, containing proteins involved mainly in cell-to-cell contact interaction, included antibodies against E-cadherin, which is mainly found in adherent junctions in tissue [21] and has been proposed as part of the signalling pathway in anoikis [22]; CD56, which is also called neural cell adhesion molecule, but has also been demonstrated to play a role in development of metastatic cells in cancer and in cell motility [23]; CD44, which is the receptor for hyaluran, a major component of the extracellular matrix, known to be involved in cell-to-cell and cell-matrix interactions [24].

**Fig. 2:**
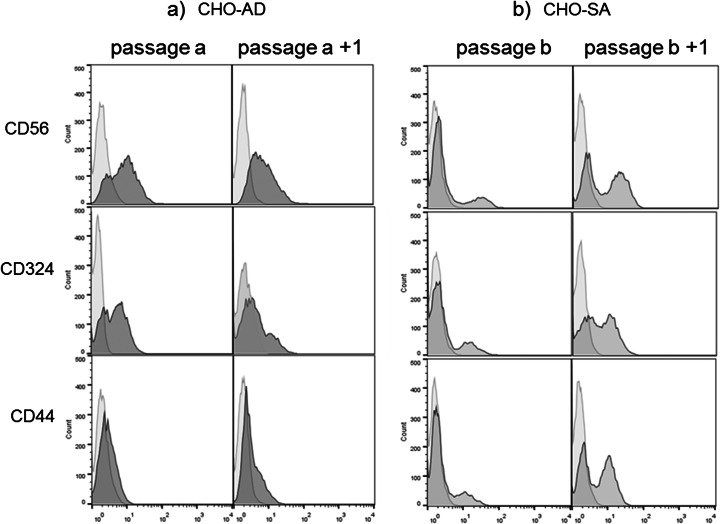
*Intracellular surface proteins—flow cytometric analysis of cell surface protein expression.* Cells were stained for cell-to-cell proteins on different days/passages. *Light grey peaks* show unstained (CD44, CD56, CD324) staining controls in histograms. *Dark grey* refers to fully stained samples. All staining were done in triplicates, but for clarity, only single examples are shown

The flow cytometric analysis of these three markers, CD56, CD44 and E-cadherin, shows a highly variable bi-modal expression in CHO-SA, with a large variation in the size of the positive subpopulation between passages (Fig. 2b). In contrast, these cell surface proteins show little variation between different passages of CHO-AD with a predominantly uni-modal distribution of expression (Fig. 2a). The existence of a CHO-SA subpopulation without expression for those markers is an indication that the expression of those proteins does not play an important role in the proliferation and survival of suspension-adapted CHO cells.

The second set of cross-reactive antibodies binds to integrins. Integrins form a group of cell surface proteins that mainly interact with the extracellular matrix or serum components in standard adherent cell culture conditions. Integrins have been demonstrated to play a role in cell adhesion, proliferation and differentiation [25, 26]. Integrin beta 1 is the main beta chain which is expressed constitutively by most mammalian cells [27]. It has been demonstrated that integrins containing the integrin beta 1 subchain bind to the RGD tripeptide motif found in most extracellular matrix components, such as fibronectin [28–30] which is also a component found in serum. As the chemically defined serum-free media used for culturing the suspension-adapted cells lacks the ligands for integrins, the integrin expression on the cell surface is of major interest in this study.

**Fig. 3:**
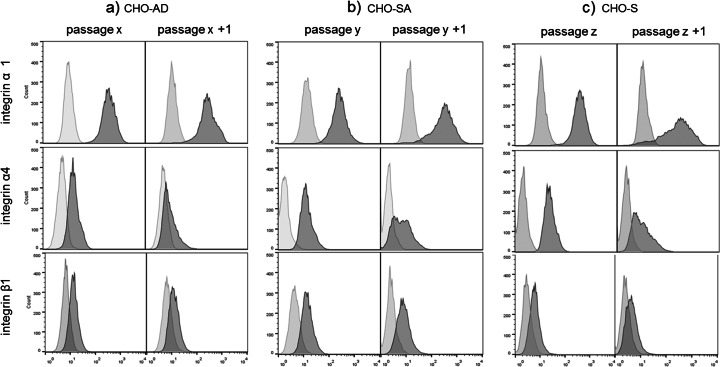
*Flow cytometric analysis of cell surface protein expression.* Cells were stained for cell-to-extracellular matrix proteins on different days/passages. *Light grey peaks* show unstained (integrin α4) or secondary only (integrin α1 and β1) staining in histograms. *Dark grey* refers to fully stained samples. All stainings were done in triplicates, but for clarity, only single examples are shown

Figure 3 shows that the integrins tested are expressed on all three cell lines. There is a clear uni-modal staining distribution occurring for CHO-SA, CHO-S and CHO-AD, which is in contrast to the results found for cell-to-cell contact proteins for CHO-SA cells; this uni-modal distribution demonstrates that integrins remain expressed on all cells within a population of suspension-adapted CHO cells beside the fact that no ligand can be found in the synthetic cell environment. There is some variation in expression level between passages which could be due to changes in expression level during cell cycle or generally due to the heterogeneity common to CHO cell lines [31]. Expression of integrins on the cell surface of two differently suspension-adapted CHO cells indicates that they play a major role in cell signalling processes and cell proliferation even in suspension-adapted CHO cells and demonstrates their importance for cell survival even in a ligand-free environment.

### Distribution of Integrins on the Cell Surface—Integrin Clustering

As demonstrated, integrins remain expressed on CHO-SA and CHO-S in the whole population over the duration of culture. A number of studies have shown that the conformational state and distribution of integrin plays a major role in how signalling occurs through them. Different integrin conformations can be linked to different ways of signalling [29, 32], the so-called inside-out and outside-in signalling. Another form of integrin activation has been described as integrin clustering where binding to ligands and hence outside-in signalling lead to the formation of integrin clusters on the cell surface [33, 34]. Considering that outside-in signalling of integrins should not be possible due to the lack of ligands within the environment, the next step in this study was to analyse whether integrin clusters can be found on the cell surface of suspension-adapted cells.

**Fig. 4:**
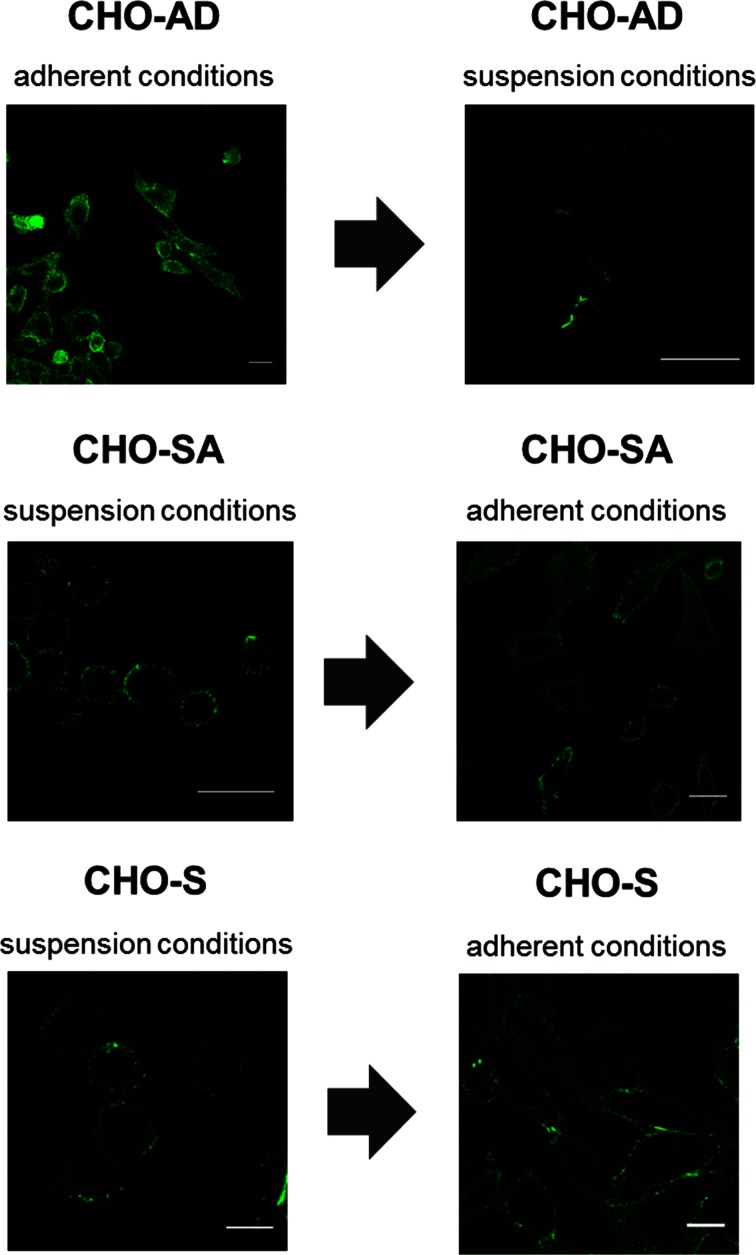
*Integrin beta 1 distribution on the cell surface of CHO-SA, CHO-AD and CHO-S.* Conformation of integrin beta 1 expression in standard conditions and changes when transferred into different culturing conditions were analysed using an inverted Zeiss LSM 510 Meta Confocal Microscope. Cells were stained after 3 days in the given culture conditions. To make dim structure visible, overall brightness for CHO-AD in suspension was increased after imaging. All *scale bars* equal 20 μm

The analysis of the integrin beta 1 staining with confocal microscopy revealed a specific clustered distribution of the protein on the cell surface of suspension-adapted CHO cells. This distribution is not seen on non-suspension-adapted CHO cells grown in suspension (Fig. 4). All cell lines showed a very similar integrin beta 1 distribution when grown adherently. Similar patterns of integrin clusters were found for integrin alpha 4 and integrin alpha 1 (data not shown), two alpha chains frequently expressed with integrin beta 1. The pattern found for integrin beta 1 on suspension-adapted CHO cells can be described as a sphere-like network of dot-like clusters covering the cell surface. Similar structures have been demonstrated to exist for activated lymphocytes [35, 36]. Considering that integrins remain expressed on suspension-adapted CHO cell beside the lack of ligands in the environment and the specific distribution on the cell surface indicating an activation of the integrins, one can assume signalling occurring within CHO-SA cells and CHO-S cells with inside-out signalling leading to integrin cluster formation. This form of signalling would be specific for suspension-adapted cell lines such as CHO-SA cells and CHO-S, as the specific distribution does not occur in CHO-AD when cultured in suspension. One of the main partners of integrin-mediated signalling is the actin cytoskeleton of cells [28], for example, integrin signalling is involved in processes such as cell division where integrin signalling helps to organise the development of the mitotic spindle [25], hence the actin cytoskeleton in CHO cells was next analysed in the differently adapted CHO cell lines.

### Actin Expression Level and Conformation in CHO Suspension Cells

**Fig. 5:**
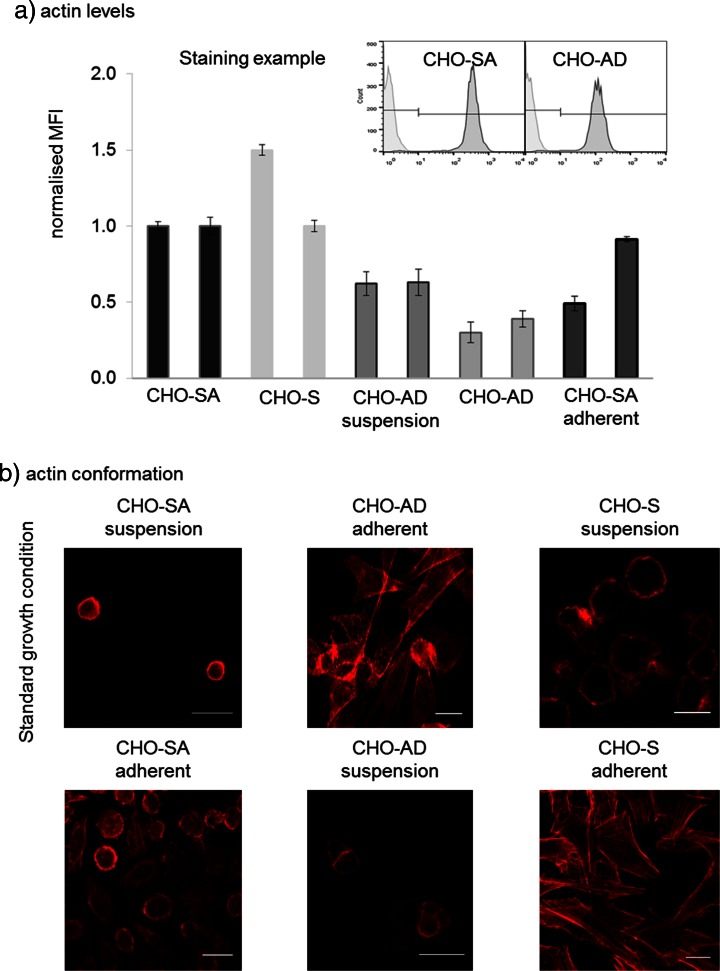
*Actin level and distribution.* **a** Example of flow cytometry data for actin level measurements and comparison of expression levels between CHO-AD, CHO-S and CHO-SA cells. Median fluorescent intensity (MFI) of CHO-SA was used to normalise the data between the randomly chosen passages. SDEV of normalised MFI is based on triplicates. **b** The actin conformation for the two cell lines in standard and reversed growth conditions. Contrast has been enhanced in all images to the same extent. All *scale bars* equal 20 μm

Figure 5a clearly shows that CHO-SA cells constitutively have a nearly twice as high level of actin expression as CHO-AD cells and that CHO-S cells show a similar or higher expression level of actin as CHO-SA. CHO-AD cells cultured in suspension also upregulate the actin content but do not reach levels comparable to CHO-SA cells whereas CHO-SA cells cultured adherently downregulate actin, but this downregulation is quite variable between different passages. The high actin content might be a requirement for survival in the suspension environment which is characterised by a high amount of shear stress due to shaking of the culture.

Confocal analysis of the actin cytoskeleton revealed that there is also a large conformational rearrangement of the cytoskeleton in suspension conditions which leads to a dense ball-like structure of the actin cytoskeleton (Fig. 5b). This is best seen in CHO-SA cells in suspension but also appears in CHO-S cells. In CHO-S, the sphere-like structure shows some ruffling which could be due to the required sample preparation steps, e.g. permeabilisation of cells. CHO-AD cells also undergo a conformational change when cultured in suspension, but the ball structure is less pronounced and the cells form clumps, as demonstrated before in Fig. 4 (top right image). The actin confirmation in adherent conditions is quite similar for all cell lines, indicating a larger flexibility allowing for more conformational changes within the CHO-SA and CHO-S cells.

### Interaction between Actin and Integrins in CHO Suspension Cells

**Fig. 6:**
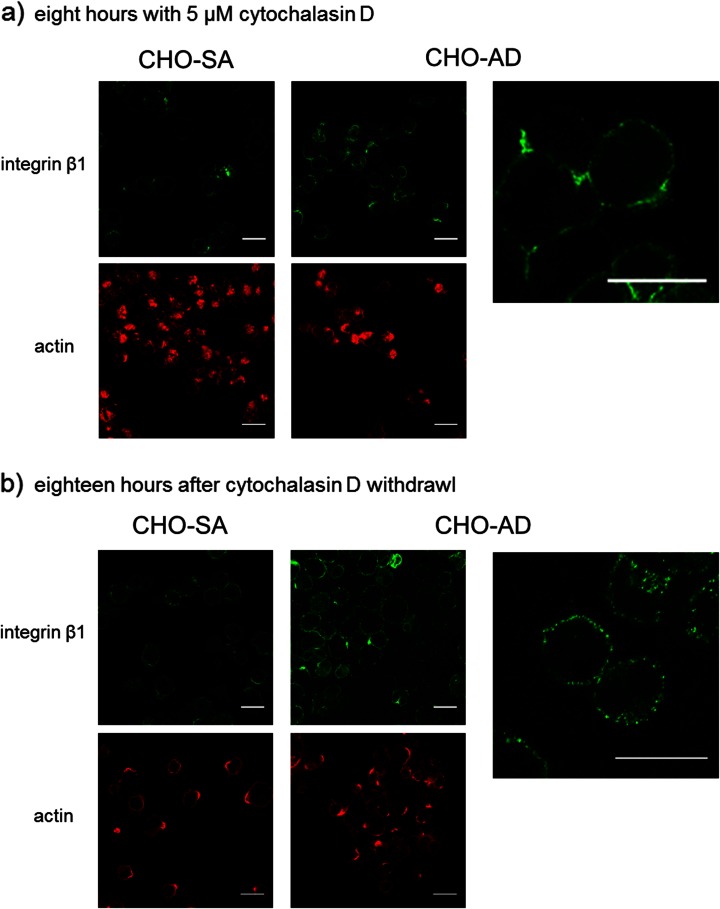
*CHO-SA and CHO-AD and cytochalasin D (CD) treatment.* After 8 h (**a**), CD was washed out and cells were grown for a period of 18 h in suspension (**b**). Cells were analysed at both stages (**a** and **b**) for integrin β1 and actin confirmation. *Scale bars* equal 20 μm

**Fig. 7:**
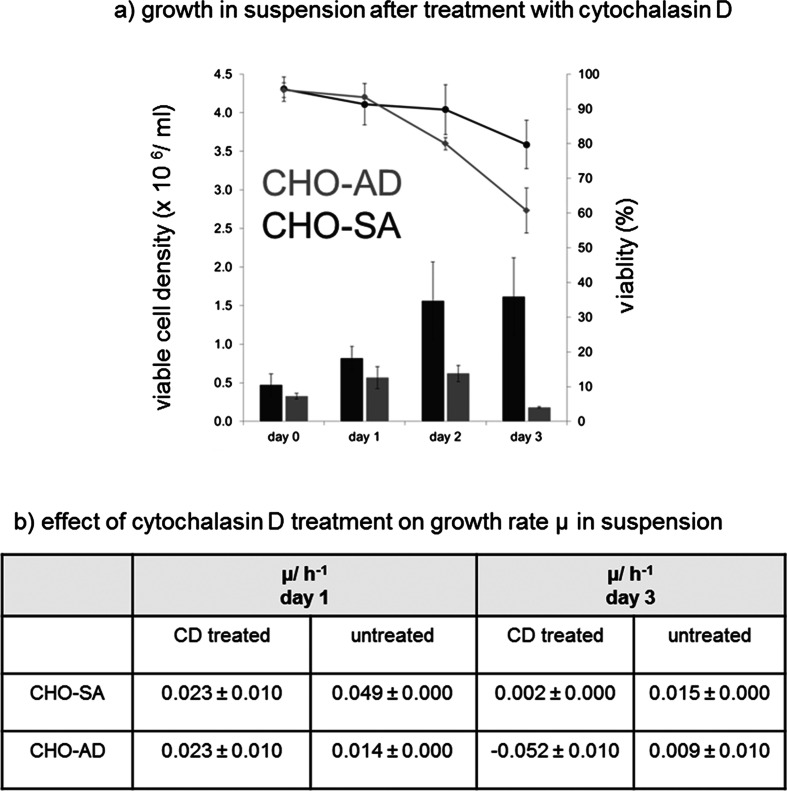
*Growth characteristics of CHO-SA and CHO-AD in suspension after treatment with cytochalasin D.* Both cell lines were initially grown adherent. After trypsin harvest and transfer into suspension culture, 5 μM CD was added to the culture. Control was treated with equivalent amount of EtOH (solvent used). After 8 h of culture, CD was washed out and cell growth was monitored for three consecutive days. **a** Results of CD-treated samples. **b** The effect of CD treatment on growth rate of the cell lines, clearly demonstrating a loss in growth due to CD treatment

The expression and distribution seen for integrin beta 1 and actin within CHO-SA and CHO-S cells indicate a highly relevant role for the interaction between these two proteins in the adaption of CHO cells to suspension. To question the interaction between integrin beta 1 and actin, cells were treated with cytochalasin D. This allows a more direct investigation of this interaction as there are numerous proteins involved in the signalling between those two proteins (Fig. 9). Cytochalasin D has been used previously to interrogate the actin cytoskeleton, study actin conformation, focal adhesion formation and integrin cytoskeleton interaction [35, 37, 38]. Cells grown in suspension and treated with cytochalasin D for 8 h show a clear disruption of the actin cytoskeleton with loss of the ball-like confirmation seen before in suspension (Fig. 6a). In addition, the net-like integrin beta 1 confirmation is lost in CHO-SA cells treated with cytochalasin D indicating a strong link between the two proteins. After withdrawal of the drug and further suspension culture for 18 h, CHO-SA cells regain their previously demonstrated confirmation for integrin beta 1 and actin. Interestingly, after the same treatment, CHO-AD cells show the same integrin beta 1 conformation in the form of a cluster covering the cell surface in a net-like fashion (Fig. 6b) as demonstrated to be typical for the CHO-SA and CHO-S cells; this indicates that a rearrangement of the integrin beta 1 has been induced by the treatment in combination with the suspension culture conditions. Uncoupling of integrin and actin has hence allowed lateral movement leading to a clustered distribution of integrin beta 1 on CHO-AD similar to what has been described before for resting lymphocytes [35]. This might also indicate a change in inside-out signalling by integrins influencing proliferation capabilities. Therefore, cells treated as described were analysed for viable cell density and growth rate after treatment for a period of 3 days (Fig. 7). As can be seen in Fig. 7a, CHO-AD cells show some growth initially but do not reach viable cell densities comparable to that of the suspension-adapted CHO-SA cells. Interestingly, at day 1, CHO-AD cells show an enhanced growth rate compared to the control cells treated with EtOH alone (Fig. 7b), indicating a positive effect of the integrin rearrangement and the underlying signalling changes on the growth capabilities. The data also demonstrates that the drug treatment in itself has a diametrical effect on the growth capabilities of the cells as can be clearly seen when comparing growth rate for CHO-SA cells: the negative effect on the cell growth set in on day 3 for CHO-SA and on day 2 for CHO-AD, indicating differences in how cell division is effected by the treatment.

### Focal Adhesion Kinase—a Linker between Integrin beta and the Actin \Cytoskeleton

**Fig. 8:**
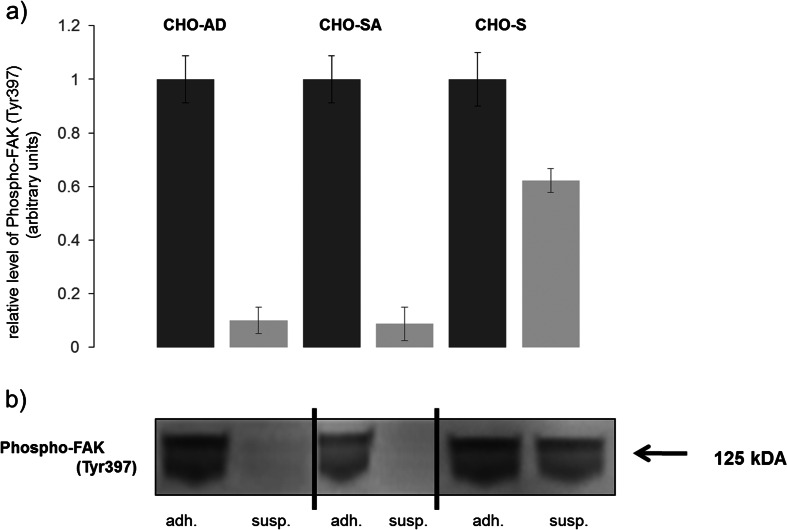
*Change of FAK phosphorylation due to change in cell culture environment.* **a** Numerical analysis of protein expression from western blot with *dark grey* showing results for adherent and *light grey* for suspension conditions. For the three experiments, expression in adherent conditions has been used to normalise the data. **b** Western blots shown are from representative experiments

Based on the data presented so far, changes in integrin inside-out signalling in conjunction with upregulation of the actin cytoskeleton appear to play a major role in suspension adaption. But it could also be a change in the outside-in signalling pathway that plays a role in suspension adaptation. Focal adhesion kinase is a linker molecule between integrin and the actin cytoskeleton which is phosphorylated at Tyr397 upon integrin activation, such as integrin clustering caused by ligand binding [28]. Hence, detection of the Tyr397 phosphorylation allows analysis of its activation status and indicates the occurrence of outside-in signalling by integrins. FAK data shows that CHO-SA and CHO-AD downregulate Tyr 397 phosphorylation of FAK when transferred from adherent into suspension conditions (Fig. 8). Downregulation of phosphorylation occurs also in CHO-S cells but to a lesser extent (approximately 40 % reduction). As CHO-SA cells grown in suspension show no phosphorylation of FAK, the clustering of integrin on the cell surface cannot be due to outside-in signalling by, for example, self-activation due to shed proteins. A form of self-activation might occur in CHO-S cells which could have acquired this capability later in the suspension adaption process; this might also promote cell clumping in larger cultures and/or at high densities. This indicates that in suspension-adapted cells, a loss of the outside-in signalling of integrins can occur beside those cells still showing clustered distribution of integrins on the cell surface.

## Conclusion

**Fig. 9:**
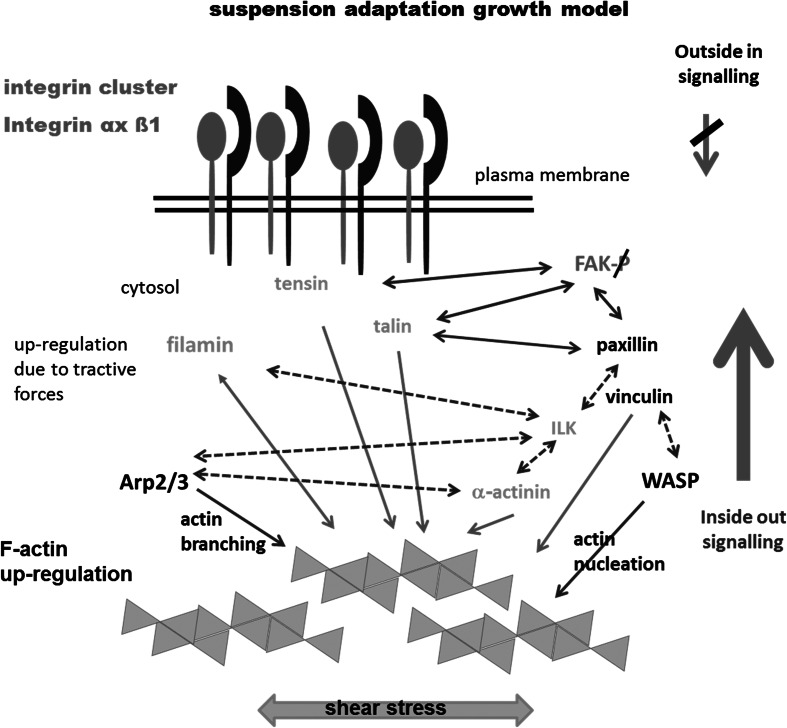
Model of integrin/actin signalling in early suspension-adapted CHO cells. Multifactorial interaction plays a role in suspension adaption. Loss of outside-in signalling, shown by loss of FAK phosphorylation, and F-actin upregulation have been demonstrated together with integrin clustering in early suspension-adapted CHO cells in this study

The data presented here indicates strongly that changes in inside-out signalling allowing clustering of integrins on the cell surface and an increase in actin filament expression are required in early successful suspension adaptation. In focal adhesion assembly, inside-out signalling in the form of tension signals from the actin filaments is catalyst for binding of vinculin and talin to the cytoplasmic integrin tail [30]. Our data demonstrates that actin filaments show an increase in expression in CHO-SA cells and tension signals will be increased due to shear stress leading to a stronger signal in this pathway. In mature focal complexes, the protein Arp2/3 links actin polymerisation to integrins via vinculin [38] leading to pronounced actin polymerisation when integrin newly engages with the extracellular matrix. Arp2/3 is responsible for actin branching and would be required to be upregulated to achieve the strong actin sheath seen in CHO-SA and CHO-S cells. Essential for successful interaction between actin and integrin are talin and integrin-linked kinase (ILK) [39] with both being capable of binding to the cytoplasmic integrin beta tail. Other proteins involved in this linkage showing an actin-binding domain are, e.g. paxillin, α-actinin and tensin [28]. So, the signalling that allows for successful proliferation in suspension will require an increase in inside-out signalling based on a variety of interaction partners and will be probably induced by shear stress and an increase in actin expression [28, 40–43]. Our model (Fig. 9) summarises and contrasts these differences in signalling between suspension-adapted CHO-SA and adherent cells CHO-AD. The data presented has demonstrated a reduction or loss in outside-in signalling as FAK phosphorylation is downregulated to different levels in the two suspension-adapted cell lines. As complete downregulation occurs in non-suspension-adapted cells when transferred into suspension, the loss might be mainly an initial effect of the serum-free suspension environment. In CHO-S cells, the phosphorylation of FAK might have been reduced to a higher extent at the beginning of the suspension adaption, and due to heterogeneity and selection pressure for high viable cell densities, clones with a higher proportion of phosphorylated FAK might have been carried forward in the generation of this cell line. The data suggests that suspension adaption will require additional transient changes in signalling, as mimicked by the cytochalasin D treatment, to allow for integrin cluster formation. As this cluster formation was demonstrated in non-adapted cells as well but was not sufficient for successful consistent proliferation in suspension, further work will require a more detailed analysis of the different signalling pathways between integrins and actin cytoskeleton and their changes during the adaptation process.

## Abbreviations

CHO: Chinese hamster ovary
PFA: Paraformaldehyde
PBS: Phosphate-buffered saline
BSA: Bovine serum albumin
EDTA: Ethylenediaminetetraacetic acid
PE: Phycoerythrin
APC: Allophycocyanin
FITC: Fluorescein isothiocyanate
FAK: Focal adhesion kinase
ILK: Integrin-linked kinase
